# GABAergic neurons in the ventrolateral periaqueductal gray mediate fentanyl withdrawal and self-administration in mice

**DOI:** 10.1038/s41386-026-02457-4

**Published:** 2026-06-05

**Authors:** Yueyi Chen, Xi Cheng, Yue Cao, Tiange Xiao, Alexander A. Chubykin, Adam Kimbrough

**Affiliations:** 1 Department of Basic Medical Sciences, College of Veterinary Medicine, Purdue University, West Lafayette, IN USA; 2 Department of Biological Sciences, Purdue University, West Lafayette, IN USA; 3 Purdue Autism Research Center, Purdue University, West Lafayette, IN USA; 4 Purdue Institute for Integrative Neuroscience, West Lafayette, IN USA; 5 Weldon School of Biomedical Engineering, Purdue University, West Lafayette, IN USA; 6 Purdue Institute of Inflammation, Immunology and Infectious Disease, West Lafayette, IN USA

**Keywords:** Reward, Motivation

## Abstract

Opioid addiction is characterized by compulsive drug seeking and use, accompanied by pain and heightened pain sensitivity (hyperalgesia) during withdrawal, yet the underlying neural mechanisms remain incompletely understood, especially for fentanyl. Using whole-brain Fos mapping and network analysis, we found that the periaqueductal gray is a central hub brain region engaged during fentanyl withdrawal, suggesting a prominent role for this region in withdrawal-associated neural signaling and warranting further examination. The ventrolateral periaqueductal gray (vlPAG) is a key site for nociceptive processing and shows robust neuronal responses during opioid withdrawal, but the role of its GABAergic neurons in fentanyl withdrawal has not been defined, and their contribution to the self-administration of drugs has not been investigated. We combined behavioral, molecular, electrophysiological, and chemogenetic approaches to examine the role of GABAergic vlPAG neurons during fentanyl withdrawal, and their contribution to withdrawal-induced hyperalgesia and fentanyl intake. During withdrawal from fentanyl, we observed increased Fos expression in GABAergic vlPAG neurons and enhanced inhibitory synaptic transmission revealed by channelrhodopsin-assisted circuit mapping, indicating increased GABAergic neuronal activity and inhibitory synaptic activity due to fentanyl use and withdrawal. Chemogenetic inhibition of GABAergic vlPAG neurons alleviated withdrawal-induced hyperalgesia. Male control mice exhibited overall escalating fentanyl self-administration across the final four intravenous self-administration sessions, whereas this escalation was not observed in males whose GABAergic vlPAG neurons were inhibited. Female mice did not exhibit a comparable effect. Together, these findings demonstrate that GABAergic vlPAG neurons are recruited during fentanyl withdrawal and contribute to withdrawal-associated nociceptive hypersensitivity. In addition, modulation of this population affects fentanyl self-administration in a sex-dependent manner. Targeting inhibitory signaling within the vlPAG may represent a strategy to alleviate withdrawal-associated hyperalgesia and limit fentanyl use.

## Introduction

The opioid crisis remains a major public health threat, with synthetic opioids such as fentanyl driving a sharp increase in overdose deaths [1]. In 2023, synthetic opioids other than methadone (primarily illicitly manufactured fentanyl) were involved in approximately 69% of all drug overdose fatalities [2]. Fentanyl, a highly potent μ‑opioid receptor agonist, has a rapid onset, high potency, and short duration of action. The high lipophilicity of fentanyl allows it to accumulate in lipid membranes and peripheral tissues, resulting in complex and prolonged pharmacokinetics. These properties increase misuse liability and contribute to particularly severe withdrawal symptoms, making dependence especially difficult to overcome [3, 4]. Fentanyl has been relatively understudied until recently, as the prominence of misuse has risen over the last decade [2]. Although much is known about the neural mechanisms that drive substance use disorders, including opioid use disorder, critical gaps remain in our understanding of the neural circuits underlying fentanyl withdrawal and fentanyl use.

Withdrawal from opioids produces symptoms including pain and hyperalgesia [5, 6]. A critically important brain region in pain processing is the periaqueductal gray (PAG), a midbrain structure enriched in opioid receptors [7]. The PAG coordinates defensive behaviors and descending pain modulation [8–10], and the ventrolateral subdivision of the PAG (vlPAG) is a key site for integrating nociceptive signaling [11, 12] and mediating opioid analgesia [10]. Within the vlPAG, GABAergic neurons have emerged as key regulators of nociception [11], aversive states [13], avoidance behavior, and contextual fear conditioning [14]. These studies firmly establish the vlPAG as a critical hub in pain-related circuits, particularly for modulating nociceptive input via endogenous and exogenous opioids.

Morphine studies have shown that opioid withdrawal increases PAG neuronal activity and induces GABAergic plasticity [15–18]. However, whether GABAergic vlPAG neurons play a causal role in withdrawal-associated behaviors remains unresolved. Moreover, the impact of fentanyl withdrawal on vlPAG inhibitory circuits has not been directly examined, and it is unknown how these neurons influence fentanyl withdrawal and fentanyl intake. Addressing this gap is critical given the lack of causal evidence for the vlPAG's role in withdrawal and drug intake associated with opioids, the distinct pharmacological properties of fentanyl, and fentanyl’s central role in the ongoing opioid crisis.

Here, we tested whether GABAergic neurons in the vlPAG are recruited during fentanyl withdrawal and contribute causally to withdrawal-associated hyperalgesia. We further examined whether activity in this population influences fentanyl self-administration. Using an osmotic minipump model of fentanyl dependence, we first established the dose and withdrawal time point associated with heightened mechanical pain sensitivity. We then used an unbiased whole-brain Fos mapping and network analysis approach to identify brain regions centrally engaged during fentanyl withdrawal, which revealed the PAG as a prominent network hub. Because whole-brain iDISCO Fos mapping does not reliably resolve PAG subregions, this systems-level analysis was used to identify candidate regions rather than specific subcircuits. Guided by this finding and prior evidence highlighting the role of GABAergic signaling within the vlPAG in nociceptive processing and opioid withdrawal [15–18], we then specifically examined whether these neurons respond to fentanyl withdrawal. To do this, we combined immunohistochemistry and channelrhodopsin-assisted circuit mapping (CRACM) to examine GABAergic neuronal recruitment and inhibitory synaptic plasticity in the vlPAG. Finally, chemogenetic inhibition of GABAergic vlPAG neurons was used to assess their role in fentanyl withdrawal-associated hyperalgesia and fentanyl use. This study identifies the role of GABAergic vlPAG neurons in mediating nociceptive dimensions of opioid withdrawal. It further shows that GABAergic neurons in the vlPAG modulate fentanyl-taking behavior. To our knowledge, this is the first work to functionally connect the vlPAG pain modulatory circuit with behavioral manifestations of fentanyl withdrawal, offering new insight into how this midbrain region contributes to the complex components of opioid dependence. This multimodal approach identified GABAergic vlPAG neurons as a critical node associated with inhibitory circuit plasticity during fentanyl withdrawal. Modulation of this circuit contributed to withdrawal-associated hyperalgesia. In addition, it modulated fentanyl intake.

## Materials and methods

### Animals

All experimental procedures were conducted in accordance with the National Institutes of Health *Guide for the Care and Use of Laboratory Animals* and approved by the Purdue University Animal Care and Use Committee. Male and female mice were used for all experiments. Mice were group-housed in a temperature- and humidity-controlled vivarium on a 12-h reverse light/dark cycle, with ad libitum access to food and water. All behavioral procedures were performed during the dark phase.

C57BL/6J mice (Jackson Laboratory, stock #000664) were used for validation of the minipump implantation fentanyl dependence model (saline, *n* = 14 [6 males, 8 females]; fentanyl 0.05 mg/kg, *n* = 13 [7 males, 6 females]; fentanyl 0.2 mg/kg, *n* = 14 [7 males, 7 females); fentanyl 0.4 mg/kg, *n* = 15 [7 males, 8 females]) and for whole-brain network analysis during naloxone-precipitated fentanyl withdrawal (*n* = 11 [5 males, 6 females]).

Vgat-IRES-Cre mice (Jackson Laboratory, stock #028862) were bred in-house and used for Fos/GAD67 immunohistochemistry (SAL, *n* = 13 [7 males, 6 females]; WD, *n* = 13 [7 males, 6 females]), chemogenetic manipulation (Sham::VEH, *n* = 10 [5 males, 5 females]; Sham::CNO, *n* = 9 [5 males, 4 females]; Gi::VEH, *n* = 10 [6 males, 4 females]; Gi::CNO, *n* = 11 [6 males, 5 females]), and intravenous self-administration (Sham, *n* = 13 [9 males, 4 females]; Gi, *n* = 15 [9 males, 6 females]) experiments.

Vgat-ChR2-EYFP mice (Jackson Laboratory, stock #014548) were bred in-house and used for CRACM electrophysiology experiments (SAL, *n* = 6 (3 males, 3 females); WD, *n* = 6 (3 males, 3 females)). Mice were between 8 and 10 weeks of age at the start of each experiment.

### Drugs

Fentanyl citrate (Cayman Chemical, #22659) was dissolved in sterile 0.9% saline, and all doses are reported as the free base equivalent. Naloxone hydrochloride (Millipore Sigma, #BP548) was dissolved in sterile 0.9% saline and administered intraperitoneally (10 mg/kg). Clozapine‑N‑oxide (CNO; TOCRIS, #6329) was dissolved in sterile 0.9% saline and administered intraperitoneally (3 mg/kg) 30 min prior to behavioral testing. Vehicle injections consisted of an equivalent volume of sterile saline.

### von Frey Test for mechanical pain sensitivity

Mechanical pain sensitivity was assessed using the von Frey test. Mice were habituated for 30 min in individual Plexiglas enclosures on an elevated wire mesh platform. Nylon monofilaments (0.04–1.4 g; Stoelting Co.) were applied to the plantar surface of the hind paw using the simplified up-down method (SUDO) [19–21]. Detailed procedures are provided in the Supplementary Methods.

### Osmotic minipump implantation and withdrawal paradigm

To induce fentanyl dependence, osmotic minipumps (Alzet LLC, model 1002) were filled with either fentanyl citrate or sterile 0.9% saline and implanted subcutaneously between the scapulae. Minipumps provide continuous, controlled drug delivery at a constant rate and are widely used to produce sustained systemic opioid exposure in rodents [22–27]. Prior studies have demonstrated that this method yields measurable and stable plasma opioid concentrations sufficient to induce canonical opioid effects, including tolerance and physical dependence [25, 28, 29]. For initial testing, to establish the ideal dose for fentanyl dependence, 0.05, 0.2, and 0.4 mg/kg were evaluated, and 0.4 mg/kg was selected for subsequent experiments. Mice were briefly anesthetized with isoflurane (3–4% induction, 1–2% maintenance) during minipump implantation. At 7 days, minipumps were surgically removed under isoflurane anesthesia to initiate spontaneous withdrawal. All mice were monitored daily throughout the infusion and withdrawal period for signs of distress or complications. For naloxone-precipitated withdrawal, at 7 days, mice were injected with naloxone to precipitate withdrawal, and brains were collected 90 min after injection.

### Whole-brain clearing and network analysis

Brains were immunolabeled and cleared using the iDISCO^+^ protocol [22, 30–33], imaged with light-sheet microscopy, and processed with ClearMap to detect Fos+ cells and register them to the Allen Mouse Brain Atlas. Fos quantification, data normalization, and criteria for excluding low-signal regions are described in the Supplementary Methods. Due to the resolution and atlas registration constraints of iDISCO^+^ and ClearMap, Fos signals were quantified at the level of the entire PAG, and individual PAG subregions (e.g., vlPAG) could not be reliably distinguished in this analysis.

Group-level correlation matrices were constructed from log_10_-transformed Fos counts, organized by major brain divisions, and converted to distance matrices for hierarchical clustering. Functional modules were defined by trimming dendrograms at 50% tree height, consistent with prior studies. Graph-theoretical metrics, including within-module degree z-score (wMDz) and participation coefficient (PC), were then calculated to identify hub regions and intermodular connectivity. Detailed pipelines, thresholding criteria, and visualization procedures are provided in the Supplementary Methods. Region abbreviations, full names, and anatomical group assignments are provided in Supplementary Table 1, and all PC and wMDz values are reported in Supplementary Table 2.

### Immunohistochemistry and imaging

To assess neuronal activity in the vlPAG, Fos/GAD67 immunohistochemistry was performed on brain sections from male and female Vgat-IRES-Cre mice treated with fentanyl or saline via osmotic minipump and collected 48 h after pump removal. Sections were immunostained using rabbit anti-c-Fos (1:500; Cell Signaling, #2250) and mouse anti-GAD67 (1:250; Millipore, #MAB5406), followed by goat anti-rabbit Alexa Fluor 488 (1:500; Thermo Fisher, A-11008) and goat anti-mouse Alexa Fluor 594 (1:500; Thermo Fisher, A32742). Images were acquired using a Zeiss LSM 880 inverted confocal microscope at 63×. Detailed immunohistochemistry procedures are provided in the Supplementary Methods.

### Electrophysiology: channelrhodopsin-assisted circuit mapping (CRACM)

To assess GABAergic synaptic transmission in the vlPAG, whole-cell voltage-clamp recordings were performed in brain slices from male and female Vgat-ChR2-EYFP mice subjected to fentanyl dependence (0.4 mg/kg/day for 7 days) or saline controls, collected 48 h after pump removal. Recordings were obtained from 6 mice per group (3 males, 3 females), with multiple cells recorded per animal (SAL: 73 cells; WD: 91 cells). Cells were sampled across animals and slices to minimize clustering within subjects. Recordings were made from visually identified vlPAG neurons using IR-DIC optics, with ChR2-EYFP expression used to guide targeting. Cells were voltage-clamped at 10 mV to isolate IPSCs. ChR2-expressing GABAergic terminals were stimulated using 470 nm light pulses (1–2 ms). Optogenetic stimulation activated vGAT-positive axon terminals within the slice, capturing both local and long-range inhibitory inputs; thus, recordings reflect overall inhibitory synaptic input rather than a presynaptic source. IPSC amplitudes were quantified and used to generate synaptic input maps. Cells with direct photocurrents or unstable baselines were excluded. Additional experimental details are provided in the Supplementary Methods and are consistent with previously described CRACM approaches [34].

### Stereotaxic surgery

Mice were anesthetized with isoflurane (3–4% induction, 1–2% maintenance) and placed in a stereotaxic frame (Kopf Instruments, Tujunga, CA, USA). For mice with active virus, AAV5-hSyn-DIO-hM4D(Gi)-mCherry (Addgene, #44362-AAV5, 5 ×10^12^ GC/mL; Gi) was bilaterally injected into the vlPAG of Vgat-IRES-Cre mice. For control mice with inactive virus, AAV5-hSyn-DIO-mCherry (Addgene, #50459-AAV5, 5 × 10^12^ GC/mL) was bilaterally injected instead. Injections were delivered using a Hamilton 7000 series 0.5 µL syringe (Hamilton, #65457-01) at a volume of 150 nL per side. Coordinates relative to bregma were AP − 4.85 mm, ML ± 0.4 mm, DV − 2.8 mm, based on prior studies [12, 35]. Virus was infused at a rate of 50 nL/min, and the syringe was left in place for an additional 5 min to allow for diffusion. Intraperitoneal injections of cefazolin (400 mg/kg) during the surgery and subcutaneous injections of carprofen (5 mg/kg) during and 3 days after the surgery were given. Mice were monitored daily and allowed to recover for a minimum of 3 weeks before behavioral testing.

### Whole-cell patch clamp recordings

To assess functional inhibition of GABAergic vlPAG neurons, whole‑cell voltage‑clamp recordings were performed in brain slices from Vgat-IRES-Cre mice injected with Gi virus into the vlPAG. Brain slices were prepared as described in the *Channelrhodopsin-Assisted Circuit Mapping* section. After baseline acquisition in ACSF, CNO (10 µM) was added to the bath and responses were recorded for 10 min.

### Intravenous catheterization and self-administration

Mice underwent jugular catheterization before fentanyl self-administration. Fentanyl IVSA was conducted in operant chambers under an FR1 schedule, in which each active lever press delivered a 2.5 µg/kg infusion followed by a 20-s timeout; inactive lever presses had no programmed consequence. Mice completed 14 consecutive 2-h sessions. On Days 11 and 14, mice received saline injections, whereas on Days 12 and 13, mice received CNO 30 min before testing. This IVSA procedure was adapted from our previous work [19]. Detailed surgical, catheter maintenance, and patency procedures are provided in the Supplementary Methods.

### Image analysis and quantification

Image analysis was performed using FIJI/ImageJ. Fos+ cells and double-labeled cells were manually counted within the vlPAG ROIs. Counts were averaged across left and right hemispheres per section. All analyses were performed blind to treatment.

### Statistical analysis

Total animal numbers and sex distribution for each experiment are reported in the Methods and corresponding figure legends. Statistical analyses were performed in GraphPad Prism (version 10.5.0). Data were tested for normality. Analyses included t-tests, one-way, two-way, three-way, or repeated-measures ANOVA, with Bonferroni *post hoc* tests as appropriate. For all experiments, sex differences were tested; unless otherwise noted, sexes were combined for analysis. Significance was defined as *p* < 0.05. Data are presented as mean ± SEM with individual data points shown where applicable.

## Results

### Forty-eight hours of fentanyl withdrawal induces robust mechanical hyperalgesia

**Fig. 1:**
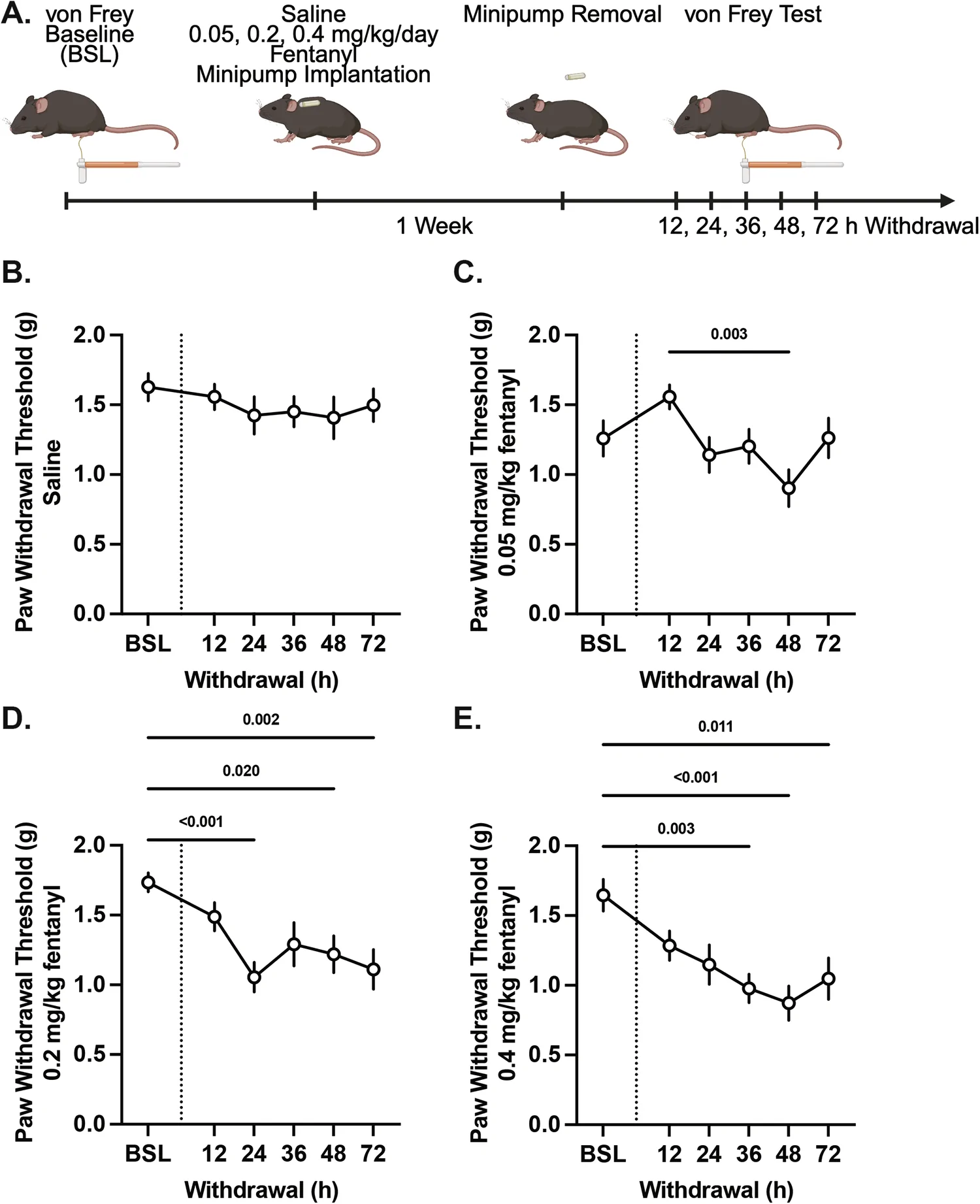
Dose- and time-dependent effects of fentanyl withdrawal on mechanical sensitivity. **A** Experimental timeline: mice were implanted with osmotic minipumps delivering saline or fentanyl (0.05, 0.2, or 0.4 mg/kg/day) for 7 days, and paw withdrawal thresholds were measured via von Frey at baseline and at 12, 24, 36, 48, and 72 h following minipump removal. **B** Saline-treated mice showed stable thresholds across time. **C** At 0.05 mg/kg fentanyl, paw withdrawal thresholds at 48 h were significantly reduced relative to 12 h fentanyl withdrawal (*F*(5,60) = 3.29, *p* = 0.011). **D** At 0.2 mg/kg fentanyl, paw withdrawal thresholds decreased significantly at 24, 48, and 72 h fentanyl withdrawal relative to baseline (*F*(5,65) = 5.48, *p* < 0.001). **E** At 0.4 mg/kg fentanyl, paw withdrawal thresholds were significantly reduced at 36–72 h fentanyl withdrawal relative to baseline (*F*(5,70) = 5.27, *p* < 0.001). Sample sizes: saline, *n* = 14 (6 males, 8 females); 0.05 mg/kg, *n* = 13 (7 males, 6 females); 0.2 mg/kg, *n* = 14 (7 males, 7 females); 0.4 mg/kg, *n* = 15 (7 males, 8 females). Data are mean ± SEM. Repeated-measures one-way ANOVA with Bonferroni *post hoc* test.

To identify the ideal dose and time point associated with robust fentanyl withdrawal-induced mechanical hyperalgesia, mice were implanted with pumps delivering saline or fentanyl (0.05, 0.2, or 0.4 mg/kg/day) and tested for mechanical pain sensitivity at 12, 24, 36, 48, and 72 h withdrawal following pump removal (Fig. 1A). Saline-treated mice showed stable thresholds across all time points. In contrast, fentanyl-exposed mice displayed dose- and time-dependent decreases in paw withdrawal thresholds. At 0.05 mg/kg, mice exhibited lower thresholds at 48 h relative to 12 h (Fig. 1C), whereas 0.2 mg/kg produced significant reductions at 24, 48, and 72 h (Fig. 1D). The 0.4 mg/kg group showed the most pronounced effect, with thresholds markedly reduced at 36–72 h (Fig. 1E). Mechanical pain sensitivity of individual animals over time is shown in Fig. S1. Based on these results, we selected 0.4 mg/kg fentanyl and the 48 h withdrawal time point for subsequent experiments.

### Whole-brain Fos network analysis reveals PAG hub role in fentanyl withdrawal

Mice were implanted with minipumps containing saline or fentanyl (0.4 mg/kg/day) for 7 days, followed by intraperitoneal saline or naloxone (10 mg/kg) injection on day 7 and tested for mechanical pain sensitivity (Fig. S2A). Fentanyl minipump- implanted mice receiving naloxone exhibited a significant decrease in paw withdrawal thresholds during withdrawal relative to baseline, whereas saline minipump-implanted mice receiving naloxone and fentanyl minipump-implanted mice receiving saline did not (Fig. S2B).

**Fig. 2:**
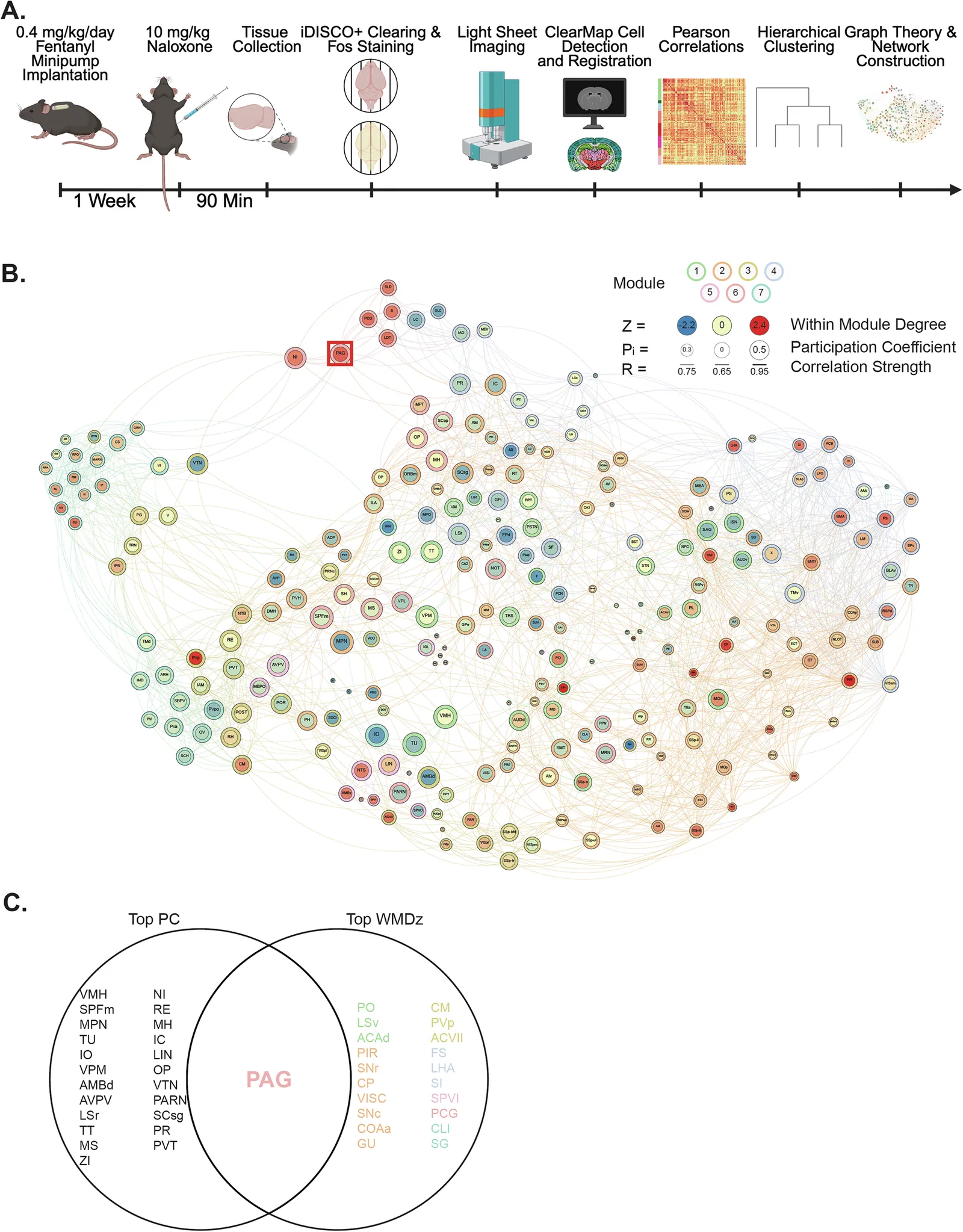
Whole-brain network reorganization during fentanyl withdrawal. **A** Experimental timeline: mice were implanted with osmotic minipumps delivering 0.4 mg/kg/day fentanyl for 7 days, followed by intraperitoneal injection of naloxone (10 mg/kg); brains were collected 90 min later and processed with iDISCO+ clearing and Fos mapping. **B** Network representation of the WD group, with nodes colored by module, with size representing PC, internal color representing WMDz, and line thickness representing correlation strength (R). The PAG is highlighted with a box. Nodes (brain regions) are represented by circles. Node size represents PC, with smaller circles indicating lower PC and larger circles indicating higher PC. The internal color of each circle represents the WMDz, ranging from dark blue (lowest) to dark red (highest). The module group is shown by the colored outline of each node. The thickness of connecting lines indicates the strength of correlation between regions, with thinner lines representing weaker correlations and thicker lines representing stronger correlations. See the figure key for schematic examples of each element. **C** Venn diagram showing the overlap between regions with top PC and top WMDz. The PAG was the only region common to both categories. Sample size: *n* = 11 (5 males, 6 females).

In a separate cohort, mice received saline or fentanyl (0.4 mg/kg/day) minipumps for 7 days, followed by intraperitoneal naloxone (10 mg/kg) injection on day 7. Brains were collected 90 min later and for iDISCO^+^ whole-brain clearing, Fos mapping, and network analysis (Fig. 2A). We calculated correlation and distance matrices based on Fos expression (Fig. S3) and used the distance matrix to define functional clusters. Region abbreviations, full names, and anatomical group assignments are provided in Supplementary Table 1. We excluded all negative correlations and applied a threshold of R = 0.75 to focus on strong connections. We used the functional clusters and correlation matrices to calculate regional network metrics, including participation coefficient (PC) and within-module degree z-score (WMDz), and constructed a fentanyl withdrawal network (Fig. 2B). All PC and WMDz values are reported in Supplementary Table 2. We then identified brain regions with the highest centrality, the top 10% of WMDz, which reflects the relative importance of a region within its own module, and the top 10% of PC, which reflects the extent to which a region connects across different modules in the network (Fig. 2C). The PAG was the only region to rank in the top 10% for both PC and WMDz, indicating its role as a central hub of the WD network. Analysis of the saline network revealed a distinct hub structure, and the PAG did not rank among the top hub regions (Fig. S4), indicating that PAG centrality was specific to the withdrawal-associated network. Based on this finding, we decided to explore the PAG further to identify its role in fentanyl withdrawal behavior and neural signaling. Because iDISCO^+^-based Fos mapping does not resolve PAG subregions, this network-level finding reflects engagement of the PAG as a whole rather than a specific subregion. Based on this result, we subsequently employed anatomically targeted approaches to examine vlPAG-specific neuronal activity and function in fentanyl withdrawal.

### Fentanyl withdrawal increases Fos expression in GABAergic vlPAG neurons

**Fig. 3:**
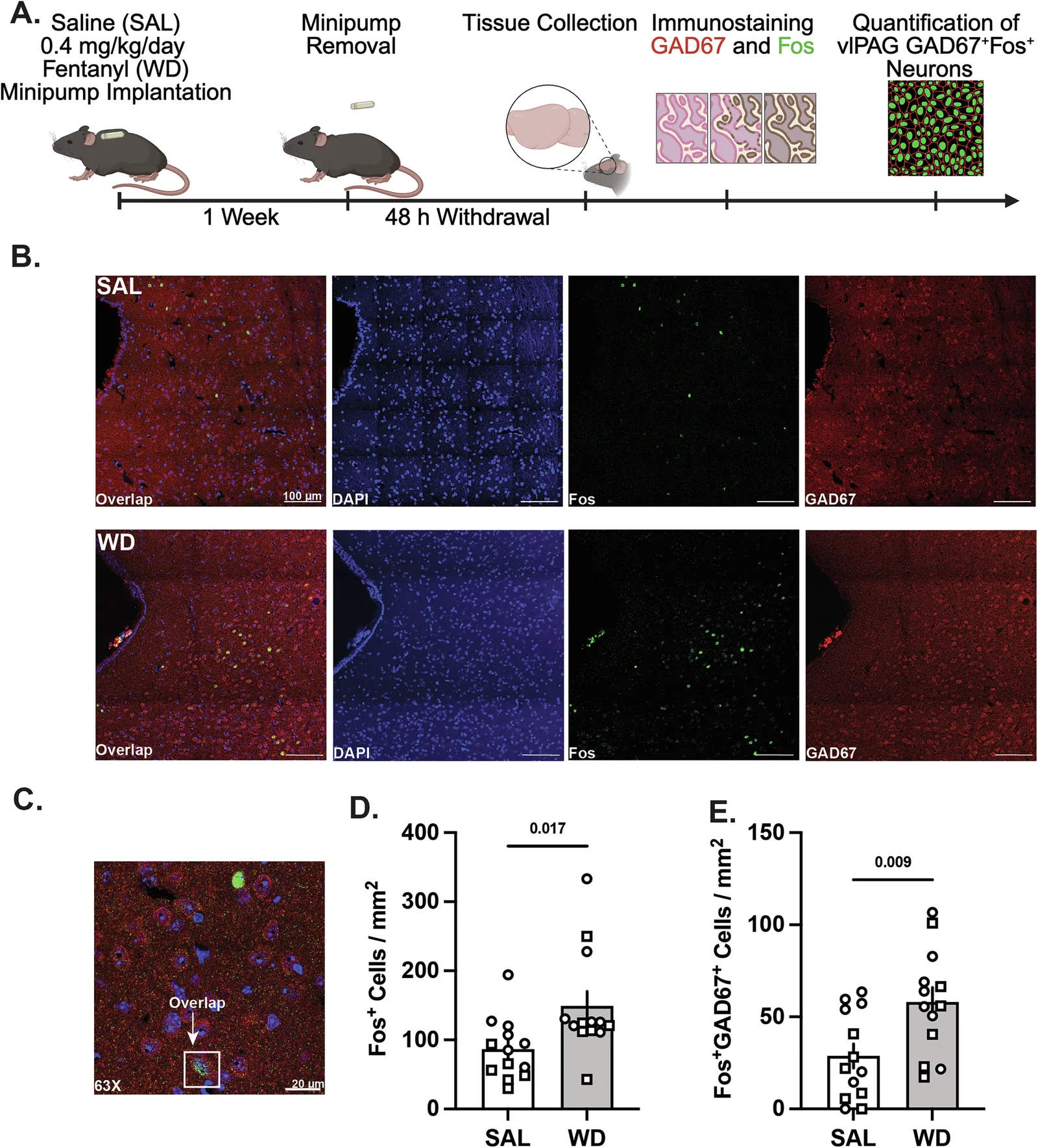
Fentanyl withdrawal increases Fos expression in GABAergic vlPAG neurons. **A** Experimental timeline: mice were implanted with osmotic minipumps delivering saline (SAL) or fentanyl (0.4 mg/kg/day; WD) for one week, and brains were collected 48 h following minipump removal (i.e., 48 h into fentanyl withdrawal). Fos and GAD67 immunoreactivity was assessed in the vlPAG. **B, C** Representative confocal images of overlap, DAPI (blue), Fos (green), GAD67 (red) staining in the vlPAG. **D** WD mice exhibited a significantly greater number of Fos^+^ neurons per mm^2^ compared to SAL controls (*t*(24) = 2.57, *p* = 0.017). **E** The number of Fos^+^GAD67^+^ double-labeled neurons per mm^2^ was higher in WD mice compared to SAL controls (*t*(24) = 2.86, *p* = 0.009). Data represent the average of the left and right vlPAG of 4 coronal sections per animal. Sample sizes: SAL, *n* = 13 (7 males, 6 females); WD, *n* = 13 (7 males, 6 females). Data are mean ± SEM. Two-tailed unpaired t-test. Circles represent males and squares represent females.

We next examined whether fentanyl withdrawal increases neuronal activity in the vlPAG. Mice received saline (SAL) or 0.4 mg/kg/day fentanyl (WD) minipumps for one week, and Fos and GAD67 expression was quantified 48 h after minipump removal (Fig. 3A). Representative staining of GAD67^+^, Fos^+^, and overlap neurons is shown in Fig. 3B, C. WD mice exhibited a significantly greater number of Fos^+^ neurons per mm^2^ in the vlPAG compared to SAL controls (Fig. 3D). To determine whether GABAergic neurons were preferentially recruited during fentanyl withdrawal, we quantified Fos^+^GAD67^+^ double-labeled cells. WD mice displayed a significantly higher number of Fos^+^GAD67^+^ neurons per mm ^2^ compared to SAL mice (Fig. 3E). Taken together, these data indicate that fentanyl withdrawal is associated with increased engagement of GABAergic vlPAG neurons in the context of an overall elevation in neuronal activity.

### Fentanyl withdrawal increases inhibitory synaptic transmission in the vlPAG

**Fig. 4:**
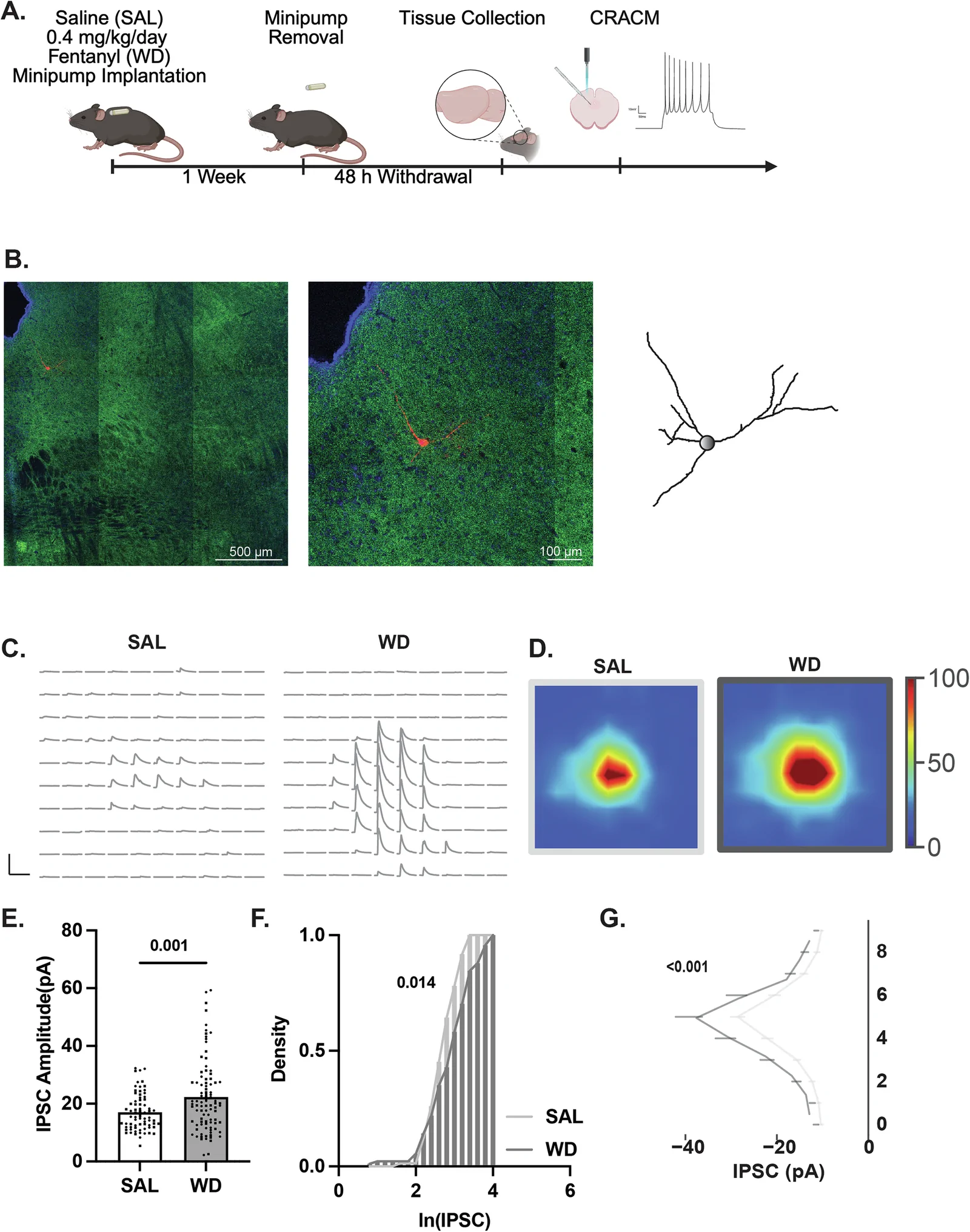
Fentanyl withdrawal enhances inhibitory synaptic transmission in the vlPAG. **A** Experimental timeline for CRACM recordings: Vgat-ChR2-EYFP mice were implanted with osmotic minipumps delivering saline (SAL) or fentanyl (0.4 mg/kg/day; WD) for 7 days, and brains were collected 48 h following minipump removal (i.e., 48 h into fentanyl withdrawal). **B** ChR2-EYFP expression in the vlPAG and representative dye-filled recorded neuron. **C** Example of CRACM_IPSC_ traces from SAL and WD mice. Scale bar: horizontal: 100 ms; vertical: .600 pA **D** CRACM heatmaps showing IPSC amplitudes across stimulation sites in SAL and WD groups. **E** Group quantification of mean IPSC amplitudes (unpaired t-test: *t*(162) = 3.23, *p* = 0.001). **F** Cumulative distribution of IPSC amplitudes (Kolmogorov–Smirnov test: *D* = 0.25, *p* = 0.014). **G** Spatial analysis of IPSC amplitudes across grid positions (two-way ANOVA: treatment, *F*(1,144) = 61.85, *p* < 0.001; grid position, *F*(1,144) = 74.97, *p* < 0.001; treatment × grid interaction, *F*(9,144) = 3.59, *p* = 0.001). Sample sizes: SAL, *n* = 73 cells from 6 mice (3 males, 3 females); WD, *n* = 91 cells from 6 mice (3 males, 3 females). Data are mean ± SEM.

To examine how fentanyl withdrawal alters inhibitory synaptic transmission in the vlPAG, we conducted CRACM recordings in Vgat-ChR2-EYFP mice 48 h into withdrawal from 7 days of minipump fentanyl exposure (0.4 mg/kg/day) or saline control (Fig. 4A). ChR2-EYFP expression was confirmed throughout the vlPAG, and targeted recordings were made from neurons located within this region (Fig. 4B).

Light-evoked inhibitory postsynaptic currents (IPSCs) recorded across a grid of stimulation sites revealed greater amplitudes and broader and more intense inhibitory input in fentanyl-withdrawn (WD) mice compared to saline-treated (SAL) controls (Fig. 4C, D). Group quantification revealed significantly higher mean IPSC amplitudes in WD animals relative to SAL (Fig. 4E), and cumulative distributions demonstrated a rightward shift in WD mice, consistent with enhanced inhibitory tone (Fig. 4F). Spatial mapping further indicated a redistribution of inhibitory inputs across the vlPAG, with WD animals exhibiting stronger and broader inhibitory responses across recording sites (Fig. 4G). To further evaluate the robustness of this effect, we performed a cell-level permutation analysis (10,000 iterations), in which group labels were randomly reassigned across all recorded cells while preserving group sizes. The observed difference exceeded 99.96% of permuted outcomes (*p* = 0.001), indicating that the effect is unlikely to arise by chance (Fig. S5). These results, paired with the observed increase in Fos expression indicate that withdrawal from fentanyl results in a significant increase in GABAergic activity within the vlPAG.

### Inhibition of GABAergic vlPAG neurons reverses fentanyl withdrawal-induced mechanical hyperalgesia and altered fentanyl self-administration in a sex-dependent manner

**Fig. 5:**
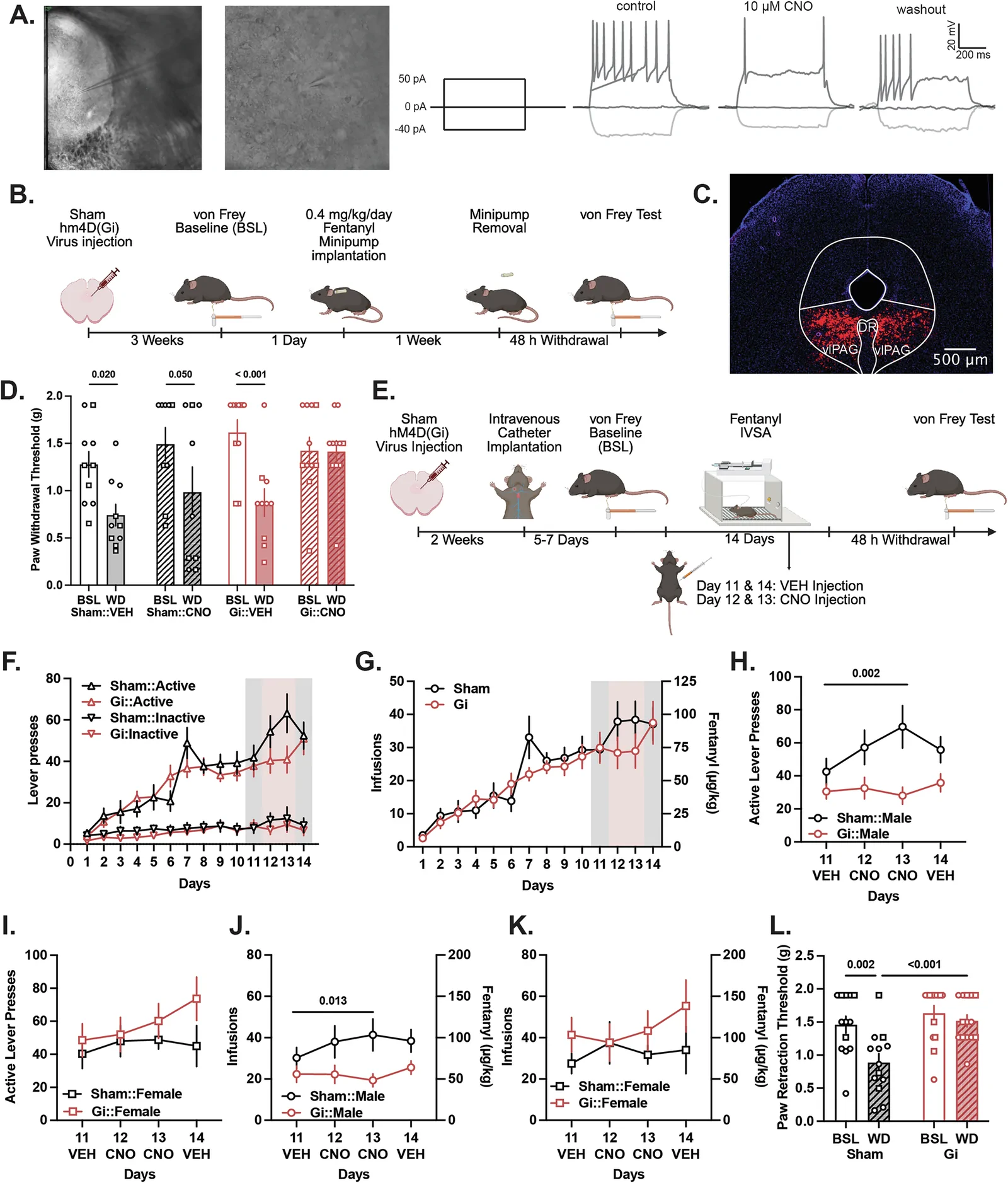
Inhibition of GABAergic vlPAG neurons reverses fentanyl withdrawal-induced mechanical hyperalgesia and altered fentanyl self-administration in a sex-dependent manner. **A** Ex vivo whole-cell recordings validated the efficacy of the chemogenetic manipulation. Bath application of CNO (10 µM) reduced action potential firing and decreased membrane excitability in GABAergic vlPAG neurons expressing Gi-DREADD (AAV-hSyn-DIO-hM4Di-mCherry). **B** Experimental design for inhibition of GABAergic vlPAG neurons in fentanyl withdrawal-associated mechanical hyperalgesia: mice were injected with Gi-DREADD (Gi) or a control virus (Sham) in the vlPAG, implanted with osmotic minipumps delivering fentanyl (0.4 mg/kg/day for 7 days), and tested for the von Frey thresholds at baseline and 48 h after minipump removal (i.e., 48 h into fentanyl withdrawal). Thirty min prior to testing, mice received intraperitoneal injections of CNO (3 mg/kg) or vehicle (VEH). This resulted in four groups: Sham::VEH (control virus, vehicle injection), Sham::CNO (control virus, CNO injection), Gi::VEH (Gi virus, vehicle injection), and Gi::CNO (Gi virus, CNO injection). **C** Representative image of virus expression in the vlPAG of GABAergic neurons expressing Gi-DREADD-mCherry. **D** Gi::CNO mice maintained withdrawal thresholds similar to baseline, whereas Sham::VEH, Sham::CNO, and Gi::VEH mice displayed significantly reduced thresholds at 48 h (three-way mixed ANOVA: withdrawal × virus × CNO, *F*(1,36) = 4.95, *p* = 0.03; withdrawal × CNO, *F*(1,36) = 5.76, *p* = 0.02; withdrawal, *F*(1,36) = 32.31, *p* < 0.001; Bonferroni *post hoc*) **E** Experimental design for inhibition of GABAergic vlPAG neurons in fentanyl self-administration: mice expressing Gi-DREADD or a control virus (Sham) in the vlPAG underwent IVSA of fentanyl for 14 days. On days 11 and 14, mice received vehicle (VEH), and on days 12 and 13, mice received CNO (3 mg/kg) 30 min prior to the session. Progressive escalation of active lever presses (**F**) and fentanyl infusions (**G**) across 14 days in both Sham and Gi mice. **H** Active lever presses of male mice over the last 4 days (two-way RM ANOVA, Virus × Days, *F*(3,48) = 3.11, *p* = 0.035; Bonferroni *post hoc*: Sham increased active lever presses). **I** Active lever presses of female mice over the last 4 days. **J** Infusions of male mice over the last 4 days (two-way RM ANOVA, Virus × Days, *F*(3,48) = 3.59, *p* = 0.020; Bonferroni *post hoc*: Sham increased active lever presses). **K** Infusions of female mice over the last 4 days. **L** Von Frey testing at baseline and 48 h following the final IVSA session (i.e., 48 h into fentanyl withdrawal) revealed reduced paw withdrawal thresholds in Sham mice but not Gi mice, with Gi mice maintaining paw withdrawal thresholds significantly higher than Sham controls (two-way RM ANOVA: withdrawal × virus, *F*(1,25) = 4.83, *p* = 0.04, withdrawal, *F*(1,25) = 11.06, *p* = 0.003; virus, *F*(1,25) = 10.04, *p* = 0.004). Sample sizes for inhibition of GABAergic vlPAG neurons in fentanyl withdrawal-associated mechanical hyperalgesia: Sham::VEH, *n* = 10 (5 males, 5 females); Sham::CNO, *n* = 9 (5 males, 4 females); Gi::VEH, *n* = 10 (6 males, 4 females); Gi::CNO, *n* = 11 (6 males, 5 females). Sample sizes for inhibition of GABAergic vlPAG neurons in fentanyl self-administration: IVSA: Sham, *n* = 13 (9 males, 4 females); Gi, *n* = 15 (9 males, 6 females). Von Frey: Sham, *n* = 13 (9 males, 4 females); Gi, *n* = 14 (8 males, 6 females). One male mouse in the Gi group was lost prior to von Frey testing. Data are mean ± SEM. Circles represent males and squares represent females.

To examine the functional effect of chemogenetic inhibition of GABAergic vlPAG neurons, we injected the vlPAG of Vgat-IRES-Cre mice with inhibitory designer receptor hM4Di (AAV5-hSyn-DIO-hM4D(Gi)-mCherry; Gi). Ex vivo whole‑cell recordings confirmed that bath application of CNO reduced action potential firing and membrane excitability in vlPAG neurons expressing Gi, validating the efficacy of the chemogenetic manipulation (Fig. 5A). The experimental design is shown in Fig. 5B. Robust expression of Gi was observed in GABAergic vlPAG neurons, confirming accurate targeting (Fig. 5C). To examine whether GABAergic vlPAG neurons contribute to withdrawal-induced mechanical hypersensitivity, we injected Gi or control virus (AAV5-hSyn-DIO-mCherry; Sham) into the vlPAG of mice. Mice were tested for baseline mechanical sensitivity using the von Frey test and then implanted with fentanyl (0.4 mg/kg) minipumps. Minipumps were removed after 7 days, and then 48 h following minipump removal (i.e., 48 h withdrawal), we administered CNO or saline and assessed mechanical sensitivity. Mice that received Gi virus injections and CNO (Gi::CNO) maintained paw withdrawal thresholds comparable to their baseline levels, whereas mice that received sham virus or saline injection (Sham::VEH, Sham::CNO, and Gi::VEH) showed significant reductions in paw withdrawal thresholds (i.e., increased mechanical sensitivity) at 48 h into withdrawal (Fig. 5D). These results indicate that inhibition of GABAergic vlPAG neurons reverses mechanical hypersensitivity during fentanyl withdrawal.

To determine whether GABAergic vlPAG neurons regulate fentanyl intake and withdrawal behaviors, we combined IVSA of fentanyl with chemogenetic inhibition. Vgat-IRES-Cre mice were injected with Gi or Sham virus in the vlPAG and tested for baseline mechanical pain sensitivity using the von Frey test (Fig. 5E). Mice underwent 14 days of fentanyl IVSA. Both Sham and Gi mice acquired fentanyl self‑administration across 14 days, with active lever presses and infusion counts progressively increasing in both groups (Fig. 5F, G). Mice showed no significant sex differences and exhibited stable active lever pressing and infusion levels across days 8–10 prior to the initiation of chemogenetic manipulation (Fig. S6A–D). When intake during the final four days of IVSA was analyzed, a significant effect of sex was detected for both active lever presses and fentanyl infusions (Fig. S6E, F); therefore, subsequent analyses were conducted with sexes separated. In male mice, a two-way ANOVA revealed a significant virus × day interaction, and *post hoc* analyses showed that Sham males increased active lever presses on day 13 (the second day of CNO injection) compared with day 11 (VEH injection), whereas Gi males did not show this increase (Fig. 5H). In contrast, female mice did not exhibit a significant virus × day interaction or changes in active lever pressing across days (Fig. 5I). A similar pattern was observed for fentanyl infusions: Sham males showed a significant increase during the CNO phase that was absent in Gi males (Fig. 5J), while no such effect was detected in females (Fig. 5K). Together, these results indicate that chemogenetic inhibition of GABAergic vlPAG neurons altered fentanyl self-administration in a sex-dependent manner. We next assessed withdrawal‑associated hyperalgesia at 48 h withdrawal from the last IVSA session. Sham mice displayed reduced paw withdrawal thresholds during fentanyl withdrawal, whereas the paw withdrawal thresholds of Gi mice were similar to their baseline levels and significantly higher than Sham controls when GABAergic vlPAG neurons were inhibited (Fig. 5L).

## Discussion

In this study, we established an osmotic minipump model of fentanyl dependence and identified an optimal dose (0.4 mg/kg/day) and withdrawal time (48 h) to assess neurobiological and behavioral signs of fentanyl withdrawal. Using the minipump model, whole-brain Fos mapping, and network analysis, we identified the PAG as a key hub brain region in the network associated with fentanyl withdrawal, suggesting the PAG may have a prominent role in fentanyl withdrawal. Using the minipump model, we found that fentanyl withdrawal significantly increased Fos expression in the vlPAG, including enhanced recruitment of GAD67^+^ GABAergic neurons. Electrophysiological recordings using CRACM revealed increased inhibitory synaptic transmission in the vlPAG. Chemogenetic inhibition of GABAergic vlPAG neurons reversed hyperalgesia associated with fentanyl withdrawal. Moreover, in a model of fentanyl IVSA, male control mice showed an overall increase in fentanyl self-administration across the last four IVSA sessions, whereas this escalation was not observed in males with GABAergic vlPAG inhibition. Overall, these data indicate that GABAergic vlPAG neurons are robustly engaged during fentanyl withdrawal, and activity in this population contributes to withdrawal-induced hyperalgesia. In addition, modulation of GABAergic vlPAG neurons alters fentanyl self-administration.

Whole-brain Fos mapping and network analysis provided insight into the systems-level neural mechanisms associated with fentanyl withdrawal. Naloxone-precipitated withdrawal was used in the whole-brain Fos mapping experiments to induce a temporally precise and synchronized withdrawal state across animals, which is critical for capturing withdrawal-associated neural activity at the systems level. To identify key regions within the neural network associated with fentanyl withdrawal, we applied two complementary centrality measures: PC, which quantifies the extent to which a brain region connects across different functional clusters, and WMDz, which assesses how strongly a brain region is connected within its own functional cluster. This unbiased, data-driven analysis highlighted the PAG as a highly central hub region, underscoring its importance in coordinating distributed brain activity during fentanyl withdrawal and warranting further examination of the causal role this region may play in withdrawal-associated neural signaling and behavior. Because iDISCO^+^-based whole-brain Fos mapping is optimized for regional-level analyses and does not resolve PAG subregional organization, this network-level finding reflects engagement of the PAG as a whole rather than specific subregions such as the vlPAG. Although these data support a withdrawal-associated PAG network signature, we cannot fully exclude the possibility that fentanyl exposure or intoxication prior to naloxone precipitation contributed to some component of the observed Fos activation, independent of withdrawal.

The goal of our network analysis was to identify regions of interest for subsequent mechanistic investigation. In follow-up experiments focused on vlPAG-specific mechanisms and behavior, we employed spontaneous withdrawal to enhance translational relevance and to assess region-specific function under conditions that more closely reflect the clinical course of opioid withdrawal. Our immunohistochemical analysis revealed that fentanyl withdrawal robustly increased Fos expression in the vlPAG, with a significant increase in Fos⁺/GAD67^+^ co-labeled neurons, indicating recruitment of GABAergic neurons. Previous studies examining naloxone-precipitated withdrawal from morphine have similarly reported elevated Fos expression in the vlPAG [36], including within GABAergic vlPAG neurons using co-staining for Fos and GAD67 [37]. Taken together, our data and prior findings reinforce the vlPAG as a critical hub region engaged during opioid withdrawal. However, most previous studies have focused on morphine, leaving the role of the vlPAG in fentanyl withdrawal largely unexplored. Our findings extend this literature by demonstrating cell-type-specific recruitment of GABAergic vlPAG neurons during withdrawal from fentanyl dependence, at a time point coinciding with heightened mechanical pain sensitivity. Importantly, increased Fos expression in GABAergic neurons does not necessarily indicate enhanced excitatory drive within the circuit as a whole, but more likely reflects increased recruitment or firing of inhibitory neurons themselves, as reported in other opioid withdrawal states [18, 38]. This approach does not distinguish between GABAergic interneurons and projection neurons within the vlPAG; therefore, the specific subpopulations contributing to withdrawal-associated Fos activation remain to be determined.

Using CRACM in Vgat-ChR2-EYFP mice, we observed increased IPSCs in GABAergic vlPAG neurons during fentanyl withdrawal, alongside a rightward shift in cumulative IPSC amplitude distributions and significant grid position × treatment interaction. Electrophysiological studies have consistently demonstrated that opioid withdrawal enhances GABAergic transmission in the PAG. In rat PAG slices, naloxone-precipitated withdrawal after chronic morphine increased GABA release through a PKA-dependent mechanism that facilitates GABAergic synaptic transmission [15]. In mice, chronic morphine similarly augmented adenylyl cyclase–cAMP–PKA signaling, rendering GABAergic terminals hyperexcitable during naloxone-precipitated withdrawal [39]. These studies align with our finding of increased inhibitory input from local GABAergic neurons during fentanyl withdrawal, and suggest that similar presynaptic mechanisms, possibly involving PKA signaling, may underlie enhanced GABA release across different opioid withdrawal models. Spontaneous opioid withdrawal has also been shown to activate a GABA transporter-1 (GAT-1)–mediated cation current in PAG neurons, increasing firing rates in a PKA-dependent manner [40]. Although we did not directly measure GAT-1 currents, the enhanced CRACM-evoked inhibitory responses we observed may reflect increased GABA release driven by mechanisms such as GAT-1–mediated depolarization. Pharmacological inhibition or genetic deletion of GAT-1 was shown to suppress morphine withdrawal-induced Fos expression and reduce somatic withdrawal signs, supporting the role of heightened GABAergic PAG activity in opioid withdrawal [38]. Importantly, the GAT-1–mediated cation current appears to result from enhanced transporter activity rather than increased transporter expression, likely through PKA-dependent trafficking or modulation. These findings complement our observation that inhibitory input strength is not uniformly elevated across the vlPAG but is instead reorganized spatially at the single-cell level, possibly reflecting regional regulation of GABAergic tone, localized GAT-1 activation, or PKA signaling heterogeneity. Additionally, inhibitory transmission changes in the vlPAG have been linked to withdrawal-related negative affect. Repeated morphine produces presynaptic and postsynaptic adaptations in GABAergic vlPAG signaling, which contributed to mechanical hyperalgesia during withdrawal [18]. Our data extend this literature by showing that fentanyl withdrawal induces functional reorganization of GABAergic microcircuits, offering a potential synaptic mechanism for the emergence of signs of negative affect. To our knowledge, no prior studies have employed CRACM to examine opioid withdrawal-induced changes in synaptic transmission. Unlike conventional IPSC recordings, which sum inputs from diverse sources, CRACM enabled us to selectively activate local vGAT+ neurons within the vlPAG and spatially map their synaptic outputs. The results revealed not only an overall enhancement of inhibitory input strength, but also a spatial redistribution of input efficacy. The significant grid position × treatment interaction suggests that inhibitory drive during withdrawal becomes reorganized rather than uniformly elevated. Together, our findings support the notion that fentanyl withdrawal functionally enhances local inhibitory microcircuits in the vlPAG. This is consistent with previous work in morphine models and extends them by demonstrating that similar adaptations occur with fentanyl, reinforcing the translational relevance of GABAergic vlPAG signaling in mediating opioid withdrawal. However, because Vgat-ChR2-EYFP mice do not permit definitive separation of local GABAergic vlPAG inputs and long-range GABAergic afferents projecting into the vlPAG, the increased inhibitory transmission observed here may reflect plasticity of local microcircuits, enhanced afferent input, or a combination of both. Future studies employing viral strategies to restrict opsin expression to GABAergic vlPAG neurons will enable a direct dissociation of local versus afferent contributions and more precisely define the source of withdrawal-induced inhibitory plasticity. In addition, we could not determine the cell-type specificity of the recorded postsynaptic neurons. Dense eYFP expression in GABAergic processes prevented reliable subtype classification, so recordings were obtained from a heterogeneous population of vlPAG neurons. While intrinsic electrophysiological properties can provide insight into cell type, our experiments were designed to assess synaptic input and were not optimized for neuronal classification. Future studies combining electrophysiology with post hoc cell-type identification will be needed to define the specific postsynaptic targets.

The vlPAG has been well established as a central modulatory hub for nociceptive processing [9], with bidirectional regulation mediated by glutamatergic and GABAergic neurons [11, 12, 35]. Inhibition of GABAergic vlPAG neurons has been shown to reduce thermal pain sensitivity without affecting mechanical pain sensitivity under normal conditions [12]. In the context of inflammatory pain, inhibition of GABAergic vlPAG neurons relieved CFA-induced mechanical hyperalgesia [11]. Our results extend these previous findings related to GABAergic vlPAG neurons and pain processing by demonstrating that chemogenetic inhibition of GABAergic vlPAG neurons reversed mechanical hyperalgesia during fentanyl withdrawal. In the baseline state, inhibition of GABAergic vlPAG neurons does not further elevate mechanical pain thresholds [11]. In contrast, under CFA-induced pathological conditions, inhibition of these neurons reduces mechanical pain sensitivity [11]. Opioid withdrawal may therefore produce a hyperalgesic state that shares features with pathological pain conditions such as CFA-induced inflammation. Our findings underscore the critical functional role of inhibitory neuronal populations within the vlPAG during opioid withdrawal. In interpreting the increased Fos expression observed in GABAergic vlPAG neurons during withdrawal, it is important to note that c-Fos expression marks only a subset of neurons that are active within a given time window, reflecting recently engaged ensembles rather than the entire neuronal population contributing to behavior. Accordingly, our chemogenetic manipulation was not intended to selectively target Fos-positive neurons, but rather to test whether GABAergic signaling within the vlPAG is necessary for withdrawal-associated hyperalgesia. While inhibition of the broader GABAergic population was sufficient to reverse hyperalgesia, it remains possible that selectively manipulating withdrawal-activated ensembles would yield distinct or more specific behavioral effects. Dissecting the relative contributions of globally active versus ensemble-specific GABAergic neurons will be an important direction for future studies.

In this study, male Sham mice showed an overall increase in fentanyl self-administration across the last four sessions, whereas this escalation was absent in males with GABAergic vlPAG inhibition. Notably, responses decreased in the final session (Day 14) in sham mice, suggesting a transient fluctuation. To determine whether this decrease reflects incomplete behavioral stabilization, we examined individual trajectories across Days 8-10. Linear regression showed that slopes were not significantly different from zero in the majority of animals, indicating that responding had largely stabilized before the manipulation and suggesting that the Day 14 decrease is unlikely to reflect incomplete behavioral stabilization before chemogenetic testing. One possible explanation is that increased fentanyl intake on preceding sessions (e.g., Days 12–13) led to a short-term reduction in motivation or compensatory regulation of intake on the following day. Behavioral variability may also increase during later stages of repeated IVSA and injection procedures due to accumulated handling or task adaptation. Although behavioral responding appeared stable and did not differ between sexes before manipulation, we cannot exclude the possibility that escalation dynamics differed by sex during Days 11–14 when chemogenetic testing occurred. Sex differences in opioid-related affective states and motivational processes have been reported previously, with males and females exhibiting divergent emotional and behavioral responses during opioid intake despite similar stress-induced relapse-related behaviors [41]. Together, these findings suggest that GABAergic vlPAG neurons regulate fentanyl intake in a sex-specific manner, potentially reflecting differences in mechanical pain processing, opioid sensitivity, or downstream circuit engagement between males and females. In both male and female Gi mice, fentanyl responding on the final vehicle day did not show a clear rebound following prior CNO treatment, suggesting that the effects of GABAergic vlPAG inhibition may extend beyond acute manipulation. While this pattern could reflect a carryover effect of CNO administration from preceding sessions, it is also possible that inhibition of GABAergic vlPAG neurons induces a more sustained shift in self-administration behavior. Although inhibition of GABAergic vlPAG neurons reduced both withdrawal-associated hyperalgesia and fentanyl intake in males, the absence of comparable effects in females indicates that additional or distinct mechanisms may govern opioid use during withdrawal in females. Overall, these data support that GABAergic vlPAG neurons are important drivers of continued opioid use, particularly in males, and highlight the importance of considering sex as a biological variable when dissecting brainstem mechanisms underlying opioid dependence.

In conclusion, our findings demonstrate that fentanyl withdrawal robustly recruits GABAergic vlPAG neurons. Chemogenetic inhibition of these neurons reversed withdrawal-induced mechanical hyperalgesia. We further found that fentanyl self-administration was altered in response to inhibition of GABAergic neurons within the vlPAG. While most previous work related to the PAG has focused on morphine, our study extends this literature by directly implicating GABAergic vlPAG neurons in fentanyl withdrawal. Furthermore, previous studies of opioids within the vlPAG have been limited in their examination of behavioral outputs of withdrawal. Importantly, our study identifies GABAergic vlPAG signaling as necessary for opioid withdrawal-associated mechanical hyperalgesia. We also found that these neurons played an important role in fentanyl consumption. These findings suggest that targeting inhibitory signaling within this region may offer therapeutic strategies to alleviate withdrawal symptoms and limit opioid use.

## Supplementary information

Supplementary Methods and Results

SupplementaryTable1_RegionAbbreviation

SupplementaryTable2_ModuleResults

## Data Availability

The data that support the findings of this study are available from the corresponding author upon reasonable request. All data necessary to evaluate the conclusions of this study are included in the main manuscript and/or the Supplementary Information.
