# Supplementary material for: GABAergic neurons in the ventrolateral periaqueductal gray mediate fentanyl withdrawal and self-administration in mice
Source: Neuropsychopharmacology. 2026 Jun 5;51(11):2184–96. doi: 10.1038/s41386-026-02457-4 (PMC13597504; doi:10.1038/s41386-026-02457-4)
Supplement: Supplementary file 1 [file 41386_2026_2457_MOESM1_ESM.docx]

**Supplementary Methods**

*von Frey Test for Mechanical Pain Sensitivity*

Testing began with a 0.16 g filament and proceeded with ascending or descending forces depending on paw retraction responses (a rapid lift or flinch following filament application), with five applications per trial. Mechanical pain sensitivity was scored as the number of paw retraction responses out of five, and data were averaged across the left and right hind paws.

*iDISCO^+^ Immunolabeling and Tissue Clearing*

Whole-brain immunolabeling and tissue clearing were performed using the iDISCO+ protocol as described in previous paper^1-5^. Briefly, brains were dehydrated through a graded methanol series (20%, 40%, 60%, 80%, and 100%; 1 h each) and incubated overnight in 33% methanol/66% dichloromethane (DCM). Samples were bleached in 5% hydrogen peroxide/methanol at 4 °C overnight, rehydrated through a descending methanol series (100%, 80%, 60%, 40%, 20%, phosphate-buffered saline (PBS); 1 h each), and permeabilized in PBS containing 0.2% Triton X-100, 20% dimethyl sulfoxide (DMSO), and 0.3 M glycine at 37 °C for 2 days. Blocking was performed in PBS with 0.2% Triton X-100, 10% DMSO, and 6% donkey serum at 37 °C for 2 days. Brains were then incubated with rabbit anti-c-Fos primary antibody (1:1000; Synaptic Systems, catalog #226003) diluted in PTwH (PBS with 0.2% Tween-20 and 10 μg/mL heparin) containing 5% DMSO and 3% donkey serum at 37 °C for 7 days. After five washes in PTwH over 24 h, samples were incubated with Alexa Fluor 647-conjugated donkey anti-rabbit IgG (1:500; Invitrogen, A31573) in PTwH with 3% donkey serum at 37 °C for 7 days, followed by another five washes in PTwH over 24 h. Brains were subsequently dehydrated through a methanol series, incubated in 100% methanol overnight, transferred into 33% methanol/66% DCM for 3 h, and washed twice in 100% DCM (15 min each). Finally, samples were cleared and stored in dibenzyl ether until imaging.

*Light-Sheet Imaging*

Cleared brains were imaged using an Ultramicroscope II (Miltenyi Biotec) equipped with a Neo sCMOS camera (Andor), a 2× objective (MVPLAPO 2×), and a SuperK EXTREME EXW-12 white light laser (NKT Photonics). Scanning was performed using three angled light sheets with a numerical aperture of 0.026 and a sheet width of 100%. Samples were imaged in the sagittal plane (right hemisphere, right side up). For detection of tissue autofluorescence, brains were scanned at 0.8× zoom (1.6× effective magnification) with a 10 µm step size using 480/525 nm excitation/emission filters, in two tiles. For Fos immunofluorescence, brains were scanned at 2× zoom (4× effective magnification) with a 6 µm step size using 640/680 nm excitation/emission filters, in twenty tiles. Images were stitched using ImSpector Pro with the TeraStitcher module and subsequently aligned across channels using affine 3D registration in ClearMap.

*Fos Quantification*

Fos-positive cells were detected using ClearMap^4,6^, following the same procedures established in our previous studies^1-3^. The autofluorescence signal captured with the 488 nm channel was used to register each brain to the Allen Mouse Brain Atlas (25 µm resolution) using a reference image. The hippocampus was used as a primary anatomical landmark to visually confirm appropriate alignment for each brain. The resulting transformation was applied to the corresponding Fos channel (640 nm) to map detected cells to annotated brain regions.

Fos-positive cells were detected using previously validated parameters^1,4-6^. The same parameters were applied to all brains to ensure consistency across experimental groups. In total, Fos^+^ cells were identified in 558 distinct regions, which were then grouped into 355 broader brain areas for interpretability by collapsing subdivisions once (e.g., individual vestibular nuclei into a single “vestibular nuclei” region), following conventions established in prior studies^1,2^. The data were further refined by excluding brain regions in which the majority of samples in a group exhibited only very sparse Fos expression (fewer than 10 cells). This step removed regions with consistently low and unreliable signal across animals, resulting in a final set of 254 regions for analysis. To account for variability in region size and baseline activity, Fos counts were log₁₀-transformed, a standard normalization approach in whole-brain activity mapping^1-3^. This normalization facilitated cross-region comparisons and downstream statistical analysis.

*Correlation and Distance Matrices*

To examine brain-wide co-activation, functional connectivity matrices were computed separately for each group using pairwise Pearson correlations across brain regions based on log_10_-transformed Fos counts. Correlations were calculated across animals within each group, capturing the co-variation of Fos activity across regions. For visualization, correlation matrices were organized by anatomical divisions of the brain defined by the Allen Mouse Brain Atlas: cortical plate, cortical subplate, striatum, pallidum, thalamus, hypothalamus, midbrain, pons and medulla. This organization did not affect^2,3^ correlation values but allowed for more interpretable visualization, as is standard practice in the field^1-3,7,8^. To further characterize the topological organization of brain-wide activity patterns, the correlation matrices were converted to distance matrices using Euclidean distance, and complete-linkage hierarchical clustering was performed. Dendrograms generated from clustering were trimmed at 50% tree height to define functional modules, consistent with prior work^1,2^. This modular structure was used to examine patterns of co-activation and network organization across experimental conditions.

*Graph-Theoretical Network Analysis*

To identify key network nodes and organizational features, we applied graph-theoretical analyses using customized Python scripts built on the Brain Connectivity Toolbox ^9^, as implemented in the bct package (https://github.com/aestrivex/bctpy). Only positively weighted connections were analyzed, and networks were constructed by thresholding Pearson correlation matrices (based on log_10_-transformed Fos counts) to include only strong positive correlations (R ≥ 0.75) based on a prior paper^2^.

Modules within each network were defined based on the modular structure derived from hierarchical clustering (see above). Two nodal metrics were then calculated for each brain region to assess its role in the network. Within-module degree z-score (WMDz) quantifies how well-connected a node is within its own module. Participation coefficient (PC) measures the extent to which a node’s connections are distributed across different modules.

These metrics, described in previous research^10^, were adapted here to handle weighted edges. A node with a high WMDz (top 10% in its module) is considered a hub within its module, whereas a high participation coefficient (top 10% in all brain regions) indicates broad intermodular connectivity.

*Network Visualization*

Functional networks were visualized using Gephi (v0.10) with the Force Atlas layout algorithm and finalized in Adobe Illustrator. Nodes were sized and colored according to connectivity metrics, and edges represented weighted interregional correlations.

*Immunohistochemistry and Imaging*

Mice were subjected to the osmotic minipump model of fentanyl dependence (0.4 mg/kg/day for 7 days) or saline treatment. Forty-eight hours after minipump removal, mice were euthanized with CO₂ and transcardially perfused with PBS followed by 4% PFA. Brains were post-fixed overnight at 4 °C and sectioned at 50 µm using a vibratome. Sections were permeabilized in 0.3% Triton X-100 in PBS for 45 min, then blocked overnight in 5% normal goat serum with 0.1% Triton X-100 in PBS. Sections were incubated overnight with rabbit anti-c-Fos (1:500, Cell Signaling, #2250) and mouse anti-GAD67 (1:250, Millipore, #MAB5406) diluted in 5% normal goat serum with 0.1% Triton X-100 in PBS, followed by washes in 0.3% Triton X-100 in PBS (4 × 30 min). Sections were then incubated overnight with goat anti-rabbit Alexa Fluor 488 (1:500, Thermo Fisher, A-11008) and goat anti-mouse Alexa Fluor 594 (1:500, Thermo Fisher, A32742) in 5% normal goat serum with 0.1% Triton X-100 in PBS, followed by PBS washes (4 × 30 min). Sections were mounted with VectaShield containing DAPI (Vector Labs). Fos/GAD67 images were acquired using a Zeiss LSM 880 inverted confocal microscope at 63×.

*Intravenous Catheterization and Self-Administration*

A catheter (VWR, i.d. 0.31 mm, o.d. 0.64 mm, #62999-850) was connected to a vascular access button (Instech Laboratories, VABM1BSM/25) and tunneled subcutaneously from the mid-scapular region to the right jugular vein. Following insertion to a depth of 1.2 cm, blood backflow was used to verify placement, and the catheter was secured with silk sutures. Catheters were flushed with 0.02 mL heparinized saline (20 U/mL) and capped. Intraperitoneal cefazolin (400 mg/kg) during surgery and subcutaneous carprofen (5 mg/kg) during surgery and for 3 days thereafter were given. Mice recovered for 5–7 days before behavioral testing. Catheters were maintained by flushing with 0.02 mL heparinized saline (20 U/mL) and cefazolin (67 mg/kg) before and after each session^11,12^. Catheter patency was assessed before training, midway through training (Day 7), and after the final session using a 15 mg/mL ketamine bolus. Patency was confirmed by immediate loss of muscle tone and ataxia within 5–10 s of infusion.

Fentanyl self-administration was conducted in operant chambers (Med Associates) under an FR1 schedule. Each active lever press delivered a 2.5 µg/kg fentanyl infusion (15 µL over 1.6 s), followed by a 20-s timeout indicated by a cue light. Inactive lever presses had no programmed consequences. Mice completed 14 consecutive 2-h sessions. On Days 11 and 14, mice received saline injections; on Days 12 and 13, they received CNO 30 min before testing.

*Electrophysiology: Channelrhodopsin-Assisted Circuit Mapping (CRACM)*

Mice were anesthetized with ketamine (0.1 mg/g) and xylazine (0.16 mg/g) and transcardially perfused with ice-cold choline-based artificial cerebrospinal fluid (choline ACSF; in mM: 110 choline chloride, 30 NaHCO₃, 25 dextrose, 11.6 ascorbic acid, 7 MgCl₂, 3.1 Na-pyruvate, 2.5 KCl, 0.5 CaCl₂; pH 7.3–7.4) until the liver appeared pale. Brains were rapidly extracted, trimmed, and mounted for sectioning. Coronal slices (300 μm) containing vlPAG were cut in ice-cold, carbogenated (95% O₂/5% CO₂) choline ACSF. Slices were transferred to standard ACSF (in mM: 124 NaCl, 26 NaHCO₃, 10 dextrose, 2.5 KCl, 2 CaCl₂, 1.25 NaH₂PO₄, 0.8 MgCl₂; pH 7.3–7.4) and incubated at 32 °C for 30 min, then held at room temperature for at least 1 h prior to recording. Slices remained viable for up to 10 h. Patch pipettes (3–6 MΩ) were filled with a cesium-based internal solution (in mM: 103 D-gluconic acid, 103 CsOH, 20 HEPES, 10 Na2-phosphocreatine, 5 QX314-Cl, 5 TEA-Cl, 4 Mg-ATP, 2.8 NaCl, 0.3 Na2-GTP, 0.2 EGTA; pH 7.2–7.3; 290 mOsm). Optogenetic stimulation was delivered through a 10 × 10 grid (67 µm spacing; Polygon 400, Mightex), and acquisition was synchronized using a Digidata 1550A and pClamp software (Molecular Devices).

**Supplementary Figure**

**Supplementary Figure 1. Dose- and time-dependent effects of fentanyl withdrawal on mechanical sensitivity.**

(A) Saline-treated mice showed stable thresholds across time.

(B) At 0.05 mg/kg fentanyl, paw withdrawal thresholds at 48 h were significantly reduced relative to 12 h fentanyl withdrawal.

(C) At 0.2 mg/kg fentanyl, paw withdrawal thresholds decreased significantly at 24, 48, and 72 h fentanyl withdrawal relative to baseline.

(D) At 0.4 mg/kg fentanyl, paw withdrawal thresholds were significantly reduced at 36–72 h fentanyl withdrawal relative to baseline.

**Supplementary Figure 2. Effect of naloxone-precipitated withdrawal on mechanical sensitivity.**

(A) Experimental timeline: mice were implanted with osmotic minipumps delivering saline or fentanyl (0.4 mg/kg/day) for 7 days, followed by an intraperitoneal naloxone challenge (10 mg/kg). Paw withdrawal thresholds were measured using the von Frey test 30 min after the challenge.

(B) Two-way ANOVA revealed a significant interaction between minipump treatment (saline vs fentanyl) and challenge (saline vs naloxone) (*F*(2,25) = 19.03, *p* < 0.001), as well as main effects of challenge (*F*(1,25) = 37.83, *p* < 0.001) and minipump treatment (*F*(2,25) = 21.84, *p* < 0.001) on paw withdrawal threshold. Saline minipump–implanted mice receiving naloxone (SN) and fentanyl minipump–implanted mice receiving saline (FS) did not differ during withdrawal (WD) compared to their own baseline (BSL). In contrast, fentanyl minipump–implanted mice receiving naloxone (FN) exhibited a significant decrease in paw withdrawal threshold during WD relative to baseline.

Sample sizes: SN (Saline+Naloxone), n = 9 (5 males, 4 females); FS (Fentanyl+Saline), n = 10 (5 males, 5 females); FN (Fentanyl+Naloxone), n = 10 (5 males, 5 females). Data are mean ± SEM. Circles represent males and squares represent females. RM two-way ANOVA with Bonferroni *post hoc* test.

**Supplementary Figure 3. Network organization of the fentanyl withdrawal brain.**

(A) Correlation matrices showing Fos expression across brain regions during fentanyl withdrawal, organized by major brain divisions.

(B) Distance matrices of hierarchical clustering reveal that the fentanyl withdrawal brain is segregated into eight functional clusters.

**Supplementary Figure 4. Network organization of the saline-naloxone brain.**

(A) Correlation matrices showing Fos expression across brain regions after naloxone injection of saline minipump-implanted mice, organized by major brain divisions.

(B) Hierarchical clustering of distance matrices reveals that the saline-naloxone brain is segregated into three functional clusters.

(C) Network representation of the saline-naloxone group, with nodes colored by module with size representing PC, internal color representing WMDz, and line thickness representing correlation strength (R). Nodes (brain regions) are represented by circles. Node size represents PC, with smaller circles indicating lower PC and larger circles indicating higher PC. The internal color of each circle represents the WMDz, ranging from dark blue (lowest) to dark red (highest). Module group is shown by the colored outline of each node. The thickness of connecting lines indicates the strength of correlation between regions, with thinner lines representing weaker correlations and thicker lines representing stronger correlations. See figure key for schematic examples of each element.

(D) Venn diagram showing the overlap between regions with top PC and top WMDz.

Sample size: n = 11 (6 males, 5 females).

**Supplementary Figure 5. Permutation test of IPSC amplitude differences.**

Distribution of mean differences (fentanyl − control) obtained from 10,000 permutations, in which group labels were randomly reassigned across all recorded cells while preserving group sizes. The red vertical line indicates the observed difference between groups. The observed effect fell within the extreme tail of the permutation distribution (p = 0.001), exceeding 99.96% of permuted outcomes, indicating that the difference is unlikely to arise by chance.

**Supplementary Figure 6. Active lever presses and infusions of male and female mice.**

(A) Active lever presses of male and female mice across days 8-10. No significant main effects of sex, virus, or day, nor any interactions, were observed.

(B) Infusions of male and female mice across days 8-10. No significant main effects of sex, virus, or day, nor any interactions, were observed.

(C) Linear regression of active lever presses. Each line represents one animal (days 8–10). Lines with a significant non-zero slope are shown in red. Only 1/28 animals (3.6%) showed a significant increase (sham male, p = 0.033), and 1/28 animals (3.6%) showed a significant decrease (Gi female, p = 0.019).

(D) Linear regression of infusions. Each line represents one animal (days 8–10); red lines indicate significant slopes. Only 4/28 animals (14.3%) showed a significant increase, including 2 sham males (p = 0.048; one near-perfect fit), 1 Gi male (p = 0.044), and 1 Gi female (near-perfect fit), indicating no robust or systematic escalation.

(E) Active lever presses of male and female mice over the last 4 days (three-way RM ANOVA, Sex × Virus, *F*(1,24) = 5.56, *p* = 0.027; Days, *F*(3,72) = 3.14, *p* = 0.030)

(F) Infusions of male and female mice over the last 4 days (three-way RM ANOVA, Sex × Virus, *F*(1,24) = 4.27, *p* = 0.050).

**Reference**

1	Ardinger, C. E., Chen, Y., Kimbrough, A., Grahame, N. J. & Lapish, C. C. Sex differences in neural networks recruited by frontloaded binge alcohol drinking. *Addict Biol* **29**, e13434 (2024). <https://doi.org:10.1111/adb.13434>

2	Kimbrough, A. *et al.* Brain-wide functional architecture remodeling by alcohol dependence and abstinence. *Proc Natl Acad Sci U S A* **117**, 2149-2159 (2020). <https://doi.org:10.1073/pnas.1909915117>

3	Kimbrough, A. *et al.* Characterization of the Brain Functional Architecture of Psychostimulant Withdrawal Using Single-Cell Whole-Brain Imaging. *eNeuro* **8** (2021). <https://doi.org:10.1523/ENEURO.0208-19.2021>

4	Renier, N. *et al.* Mapping of Brain Activity by Automated Volume Analysis of Immediate Early Genes. *Cell* **165**, 1789-1802 (2016). <https://doi.org:10.1016/j.cell.2016.05.007>

5	Renier, N. *et al.* iDISCO: a simple, rapid method to immunolabel large tissue samples for volume imaging. *Cell* **159**, 896-910 (2014). <https://doi.org:10.1016/j.cell.2014.10.010>

6	Kirst, C. *et al.* Mapping the Fine-Scale Organization and Plasticity of the Brain Vasculature. *Cell* **180**, 780-795 e725 (2020). <https://doi.org:10.1016/j.cell.2020.01.028>

7	Carrette, L. L. G. *et al.* Hyperconnectivity of Two Separate Long-Range Cholinergic Systems Contributes to the Reorganization of the Brain Functional Connectivity during Nicotine Withdrawal in Male Mice. *eNeuro* **10** (2023). <https://doi.org:10.1523/ENEURO.0019-23.2023>

8	Carrette, L. L. G. *et al.* Antagonists of the stress and opioid systems restore the functional connectivity of the prefrontal cortex during alcohol withdrawal through divergent mechanisms. *bioRxiv* (2024). <https://doi.org:10.1101/2023.09.30.560339>

9	Rubinov, M. & Sporns, O. Complex network measures of brain connectivity: uses and interpretations. *Neuroimage* **52**, 1059-1069 (2010). <https://doi.org:10.1016/j.neuroimage.2009.10.003>

10	Guimera, R. & Nunes Amaral, L. A. Functional cartography of complex metabolic networks. *Nature* **433**, 895-900 (2005). <https://doi.org:10.1038/nature03288>

11	Chen, Y., Xiao, T. & Kimbrough, A. Escalation of intravenous fentanyl self-administration and assessment of withdrawal behavior in male and female mice. *Psychopharmacology (Berl)* **242**, 1419-1435 (2025). <https://doi.org:10.1007/s00213-024-06739-x>

12	Towers, E. B., Tunstall, B. J., McCracken, M. L., Vendruscolo, L. F. & Koob, G. F. Male and female mice develop escalation of heroin intake and dependence following extended access. *Neuropharmacology* **151**, 189-194 (2019). <https://doi.org:10.1016/j.neuropharm.2019.03.019>
